# Hippocampal Neuronal Loss in Infant Macaques Orally Infected with Virulent Simian Immunodeficiency Virus (SIV)

**DOI:** 10.3390/brainsci7040040

**Published:** 2017-04-10

**Authors:** Heather Carryl, Koen K. A. Van Rompay, Kristina De Paris, Mark W. Burke

**Affiliations:** 1 Department of Physiology and Biophysics, Howard University, Washington, DC 20059, USA; heather.m.carryl@bison.howard.edu; 2 California National Primate Research Center, University of California Davis, Davis, CA 95616, USA; kkvanrompay@ucdavis.edu; 3 Department of Microbiology and Immunology, University of North Carolina, Chapel Hill, NC 27599, USA; abelk@med.unc.edu

**Keywords:** pediatric human immunodeficiency virus (HIV), hippocampus, design-based stereology, non-human primate, simian immunodeficiency virus (SIV)

## Abstract

The neurological impact of Human Immunodeficiency Virus (HIV) on children includes loss of brain growth, motor abnormalities and cognitive dysfunction. Despite early antiretroviral treatment (ART) intervention to suppress viral load, neurological consequences of perinatal HIV-1 infection persist. Utilizing the pediatric simian immunodeficiency virus (SIV) infection model, we tested the hypothesis that early-life SIV infection depletes neuronal population in the hippocampus. A total of 22 ART-naïve infant rhesus macaques (*Macaca mulatta*) from previous studies were retrospectively analyzed. Infant macaques were either intravenously (IV) inoculated with highly virulent SIVmac251 at ~1 week of age and monitored for 6–10 weeks, or orally challenged with SIVmac251 from week 9 of age onwards with a monitoring period of 10–23 weeks post-infection (19–34 weeks of age), and SIV-uninfected controls were euthanized at 16–17 weeks of age. We have previously reported that the IV SIVmac251-infected neonatal macaques (Group 1) displayed a 42% neuronal reduction throughout the hippocampal cornu ammonis (CA) fields. The orally-infected infant macaques displayed a 75% neuronal reduction in the CA1 region compared to controls and 54% fewer neurons than IV SIV infants. The CA2 region showed a similar pattern, with a 67% reduction between orally-infected SIV subjects and controls and a 40% difference between IV-and orally-infected SIV groups. In the CA3 region, there were no significant differences between these groups, however both SIV-infected groups had significantly fewer pyramidal neurons than control subjects. There was no correlation between plasma viral load and neuronal populations in any of the CA fields. The loss of hippocampal neurons may contribute to the rapid neurocognitive decline associated with pediatric HIV infection. While each subfield showed vulnerability to SIV infection, the CA1 and CA2 subregions demonstrated a potentially enhanced vulnerability to pediatric SIV infection. These data underscore the need for early diagnosis and treatment, including therapeutics targeting the central nervous system (CNS).

## 1. Introduction

Globally, there are over 3.2 million children under the age of 15 currently living with human immunodeficiency virus (HIV), with an estimated one new diagnosis every 2 min [1,2]. Mother-to-child-transmission (MTCT) via pregnancy, delivery or breast-feeding accounts for 15%–25% of new infections [3]. Due to the success of antiretroviral therapy (ART) for pregnant HIV-infected women, perinatal HIV-infection has been drastically reduced. In the United States, less than 200 babies are infected with HIV annually, due to the use of ART and the avoidance of breastfeeding [4]. In contrast, in under-resourced parts of the world (e.g., Sub-Saharan Africa), ART access still varies widely from country to country [5], and exclusive breastfeeding is necessary to reduce the risk of mortality due to malnutrition or non-HIV-1 illnesses such as respiratory infections and diarrhea [6,7,8]. Therefore, about 15% of HIV MTCT cases now occur as a result of breastfeeding [3]. Furthermore, even during ART [9], HIV-1 can persist in breastmilk. Although ART reduces the risk of MTCT of HIV in utero and peri-partum, adequate access and adherence to ART throughout the period of breastfeeding [10,11], mixed feeding/breastfeeding practices, and undiagnosed acute HIV infection of breast-feeding mothers, limit its effectiveness [12,13,14]; accordingly, in resource-poor areas, HIV MTCT rates remain high [15]. Another success of ART has been the increased rate of survival of HIV-infected children [16,17,18]. The survival of these HIV-infected infants into adolescence and adulthood poses a unique set challenges [17,19,20], as these HIV-1 positive children present with a high prevalence of cognitive and neurodevelopmental deficits [21,22,23]. 

The neurological impact of HIV-1 infection includes loss of brain growth, motor abnormalities and cognitive dysfunction [24]. Data suggests a high prevalence of encephalopathy, hyperreflexia, delayed milestones, quadriparesis, spasticity, and microcephaly as a result of perinatal HIV-1 infection [25]. In ART-naive children, high viral load at 6 months of age was associated with a more severe neurodevelopmental dysfunction at 12 months compared to children with lower viral load [26]. A similar pattern has been reported following a course of a 48-week ART treatment [27]. Poor cognitive performance and reduced cortical gray matter volume in perinatally HIV-infected adolescents was also associated with higher peak viral load [28]. Despite the ability of early ART intervention to partially alleviate neurological consequences of perinatal HIV-1 infection, deficits persist even after viral load suppression [22,27,29,30]. Diffusion tensor imaging has been indicative of reduced radial diffusivity, which is suggestive of demyelination [31]. This finding is consistent with scarce reports of multiple-sclerosis-like disorders in HIV-1-infected children [32]. There are notable differences between HIV-infected children and adults. HIV-associated dementia, a commonly occurring neurological disorder in HIV-infected adults, is not typically observed in HIV-infected children. While HIV has not been shown to infect neurons, cognitive impairments consistent with pathological evidence of neuronal damage, such as synaptic loss and dendritic simplification, have been reported [33]. In adult subjects, elevations in cerebrospinal fluid and low molecular weight neurofilament core chains (CSF NFL) have been suggested to serve as a sensitive surrogate marker of neuronal damage, supported also by chronic changes to white matter in various other neurodegenerative diseases, such as Alzheimer’s [34]. However, CSF NFL levels are elevated in early and later stages of HIV-1 infection in adults with and without neurocognitive impairment [35,36]. CSF NFL levels also decrease with ART initiation and increase with ART interruption [37]. Analogous data are currently missing for the pediatric HIV-1 population.

Animal models may provide us with the opportunity to elucidate the neurological effects of pediatric infection [38]. Small animal models such as rats and mice are an accessible and practical substitute method of investigating neuropathogenic mechanisms of pediatric HIV-1; but there are a few noted limitations with rodent models. One obvious limitation is that rats and mice are not the natural hosts of HIV-1 and are not susceptible to HIV-1 infection, therefore they do not develop the disease [39]. Trans-activator of transcription (Tat)/Envelope glycoprotein (gp120) intracranial administration in rodents has been used to define certain aspects of neuronal damage related to pediatric HIV-1 infection [40,41]. Rodent models have also shown that gp120 and Tat_1-72_ are involved in the neuropathophysiology of HIV-1 infection, with notable susceptibility of the hippocampus to the neurotoxic cascade of HIV-1 proteins [40,42]. The administration of Tat_1-72_ was found to impair spatial memory in adolescence [40]. However, neonatal intrahippocampal gp120 administration transitorily alters sensory-motor function [43,44]. Design based stereology demonstrated that neonatal intrahippocampal Tat_1-72_ and gp120 administration resulted in detrimental and regionally selective cell loss within the hippocampus [40]. The protein gp120 was found to reduce the neuronal population within CA2 and CA3 regions of the neonatal hippocampus while intrahippocampal Tat administration reduced neuronal populations in the CA2/3 subfields and the hilus of the dentate gyrus [40]. 

Additional research findings supported the hypothesis that Tat plays a significant role in pediatric HIV-1 neuropathogenesis and the overall psychological deterioration that is observed in HIV-1 infected children [42]. Results from in vitro cell culture studies indicate that the release of Tat occurs when the rate of cell death is low (as seen during infection), or when cell death is absent (as seen after transfection), and Tat expression is high [45]. In the former study, the highest expression of Tat was observed 48 hours post-transfection with 5%–10% of transfected cells expressing Tat. Results implied that cell growth requires a lower concentration of extracellular Tat than the transactivating effect [45]. A Tat concentration of 100 ng/mL or higher did not produce any growth stimulation, however at concentrations of 0.05 to 50 ng/mL, with peak activity at 0.1 to 1 ng/mL, growth stimulation was observed [45]. Although Tat has been shown to be a part of the neuropathology in perinatal HIV-1 infection, the question whether neuropathology is related to viral load remains unanswered. In adults, neuronal loss can be related to vial load, however, a similar link has not been established in children who experience HIV pathogenesis at a time of neuronal development. 

The simian immunodeficiency virus (SIV) infection model in macaques offers a valid alternative because SIV and HIV-1 have similar pathogenesis, including infection of CD4+T cells and macrophages, immune suppression, disease progression, neurological complications in juvenile and adult primates, and routes of transmission [46]. MTCT of SIV can occur by the same routes in monkeys and humans [47]. In a vertical infection model in pigtailed macaques (*Macaca nemestrina*), offspring of dams that were intravenously (IV)-inoculated with HIV-2287 during the third trimester displayed significant motor and cognitive delays that were correlated with cluster of differentiation 4 (CD4+) T lymphocyte cell counts at birth [48]. We have previously shown that neonatal rhesus macaques infected with SIVmac251 (IV) showed significant neuronal reductions in the hippocampus, loss of immature neurons and gross demylination [38,49]. Here, we expand on these data and investigate the effects of oral SIVmac251 infection at nine weeks of age, simulating oral HIV-1 acquisition by breastfeeding human infants, on hippocampal neuronal populations.

## 2. Materials and Methods

### 2.1. Subjects

The brain samples used in the current study were all collected as part of previously conducted studies [50,51,52,53]. The infant rhesus macaques (*Macacca mulatta*) in those former studies were nursery-reared in pairs at the California National Primate Research Center (CNPRC) in accordance to American Association for Accreditation of Laboratory Animal Care Standards, with all protocols being approved by the UC Davis Institutional Animal Care and Use Committee. All procedures were performed under ketamine-HCl anesthesia (10 mg/kg i.m.; Parke-Davis, Morris Plains, NC).

The 22 infant rhesus macaques consisted of SIVmac251 IV-inoculated neonates (*n* = 3) with a monitoring time of 6–10 weeks (hippocampal neuronal counts for this group were previously reported; see [49]). Infant macaques in Groups 2A-C were part of a vaccine study [50,51] and received a weekly oral (PO) low-dose SIVmac251 inoculation regimen (5000 tissue culture infective dose (TCID) 50 per dose) starting at ~ week 9 of age until infected; then infants were monitored for 10–23 weeks of infection (Table 1). It should be noted that the animals in Groups 2B and 2C had received different SIV vaccine regimens prior to oral SIV challenge (Table 1) [50,51]. Blood samples were drawn on a weekly basis for the PO groups and plasma SIV levels were analyzed [50,51]. Infants in Group 3 represented SIV-naïve control infant macaques (*n* = 4) that were euthanized between 16–17 weeks of age. 

### 2.2. Brain Preservation and Histology

Upon euthanasia, brains were extracted, post-fixed in 10% buffered formalin phosphate, blocked into 1-cm slabs in the coronal plane, cryoprotected in 30% buffered sucrose, and frozen at −80 °C until further processing. Ten parallel series of coronal sections (50 μm) were obtained from each animal, the first series being Nissl stained with cresyl-violet for design-based stereology, with the remaining sections placed in antigen preserve for future studies [49]. 

Systematic sections through the hippocampus were Nissl stained and hippocampal pyramidal neurons were quantified using design-based stereology. Although there is an age variance between the groups, hippocampal neuronal populations through the CA fields has been shown to be stable from birth through at least two years of age [54,55]. SIVmac251 was obtained from the Analytical and Resource Core at CNPRC. Plasma viral loads were quantified by real-time reverse transcription- polymerase chain reaction (RT-PCR) [50,56].

### 2.3. Design-Based Stereology

Total estimation of the pyramidal neuronal population of the hippocampus cornu ammonis fields (CA1–3) was achieved using the optical fractionator method. Sampling parameters are similar to previously described methods [49] where the topography (4x objective) and superimposed counting frames (dissectors 16,000 μm^3^; plan fluor oil-immersion 100x numerical aperture (N.A.) = 1.3) were generated through the MicroBrightField StereoInvestigator program (Williston, VT, USA). The stereological parameters are the same as previously used for Group 1 [49] where every 20th section was selected, a standard scan x-y grid was employed (500 μm × 500 μm, 250 μm × 250 μm, and 350 μm × 350 μm for CA1, CA2 and CA3, respectively), and the total estimation of pyramidal neuronal numbers (N) was calculated by the following equation:

N = ssf^1^ × asf^1^ × tsf^1^ × ∑Q^−^

where ssf is the section-sampling fraction, asf is the area-sampling fraction, tsf is the thickness-sampling fraction, and ΣQ^−^ is total number of objects of interest counted. The average coefficient of error (CE) for total number of neurons was determined to assess reliability of measurement was less than 0.12 for all subjects.

### 2.4. Statistical Analysis

Due to the small group sizes, statistical differences were determined only between two groups at a time applying both one-tailed [49] and two-tailed non-parametric Mann–Whitney *U* test of significance using the GraphPad Prism V7, and InStat3 programs (La Jolla, California, CA, USA). Correlation analyses between viremia and neuronal data were performed using the Spearman Rank test. Significance was determined as *p* < 0.05. Viral loads were log transformed and then calculated as area under the curve (AUC) as a way to express overall exposure to avoid acute peaks or multiple peaks in viremia from carrying a relatively larger weight (i.e., skewing the data) than for example if a subject has no peaks but a persistent intermediate viremia. 

## 3. Results

**Figure 1:**
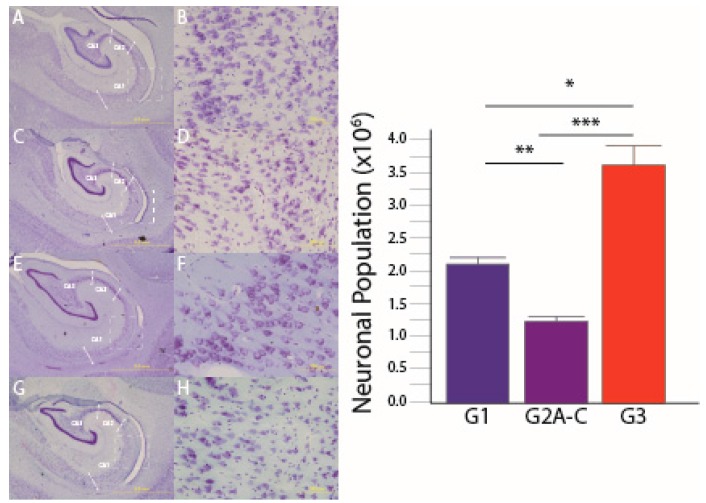
Reduced hippocampal neuronal populations: Compared to controls, Group 3 (**A**,**B**), intravenous (IV) SIV-infected neonatal macaques, Group 1, (**C**,**D**) displayed a 42% neuronal reduction throughout the hippocampal cornu ammonis (CA) fields. The orally-infected infant macaques Group 2B (**E**,**F**) and Group 2A (**G**,**H**) displayed a 70% neuronal reduction compared to controls and 47% fewer neurons than Group 1 infants. Graphs represent combined data across the different CA subfields. Magnifications A, C, E, G 1.25x (scale bar = 5 mm), and 20x B, D, F, H (scale bar = 200 μm). * *p* = 0.029, ** *p* = 0.005, *** *p* = 0.0005. One-tailed test between G3 and G1 [49] all other comparisons were two-tailed tests.

**Figure 2:**
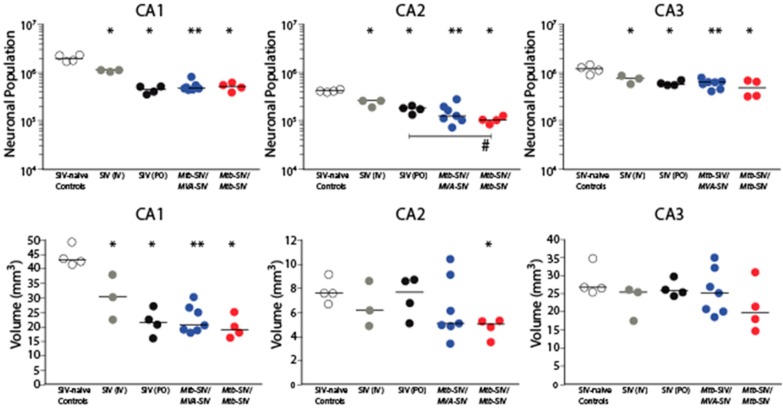
Reduced hippocampal neuronal populations and volumes. Groups 2A–C display significantly lower neuronal populations compared to Group 3. Volume differences were restricted to the cornus ammonis (CA)1 and CA2 subfields. *p* values reflect differences between each of the treated groups (G2A–C and G3) and the simian immunodeficiency virus-naïve (SIV-naïve) control group (G1). * *p* = 0.029, ** *p* = 0.006 except # *p* = 0.03 which is a comparison between Groups 2A and 2C; bar in the middle of the data points represents mean. One-tailed test between G3 and G1 [49] all other comparisons were two-tailed tests.

We have previously reported that IV SIVmac251-infected infants (Group 1) displayed a 42% neuronal reduction throughout the hippocampal CA fields [49]. Here, we show that orally SIVmac251-infected infants (Group 2A) subjects showed a significant neuronal reduction compared to SIV-naïve controls in all three CA regions (*p* = 0.0286; Figure 1 and Figure 2). It should be noted though that the duration of SIV infection in Group 2 animals was slightly longer (10–12 weeks) compared to Group 1 SIV-infected macaques (7–10 weeks). Vaccinated and orally SIV-challenged infant macaques in Groups 2B and 2C also displayed a significant neuronal reduction in all three CA regions compared to controls (Figure 2). In regions CA1 and CA3, these neuronal reductions of Group 2B and Group 2C animals were comparable to those observed in non-vaccinated orally SIV-infected macaques of Group 2A (Figure 2). However, in the CA2 region, the neuronal reduction of infant macaques that had received two doses of the *Mycobacterium tuberculosis* (*Mtb*)-SIV vaccine prior to SIV infection had an even more severe neuronal reduction than non-vaccinated SIV-infected Group 2 macaques (Figure 2). 

**Figure 3:**
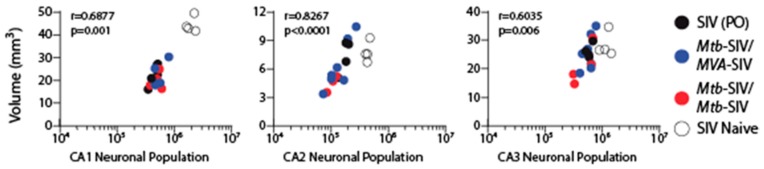
Neuronal population and volume correlation. There was a significant correlation between neuronal populations and volume in the cornu ammonis (CA) subfield 1 (*p* = 0.0011), CA2 (*p* < 0.0001) and CA3 (*p* < 0.0062) hippocampal fields.

Volume differences were found only in the CA1 and CA2, but not the CA3 region between the various groups (Figure 2). Both CA1 and CA2 volumes were significantly lower in Groups 1 and 2A–C compared to controls (Group 2) (Figure 2). The volume in infant macaques orally infected with SIVmac251 (Group 2A) was comparable to that observed in Group 2B and Group 2C infants that were vaccinated prior to SIV infection (Figure 2). There was a significant correlation between volume and neuronal population (Figure 3).

**Figure 4:**
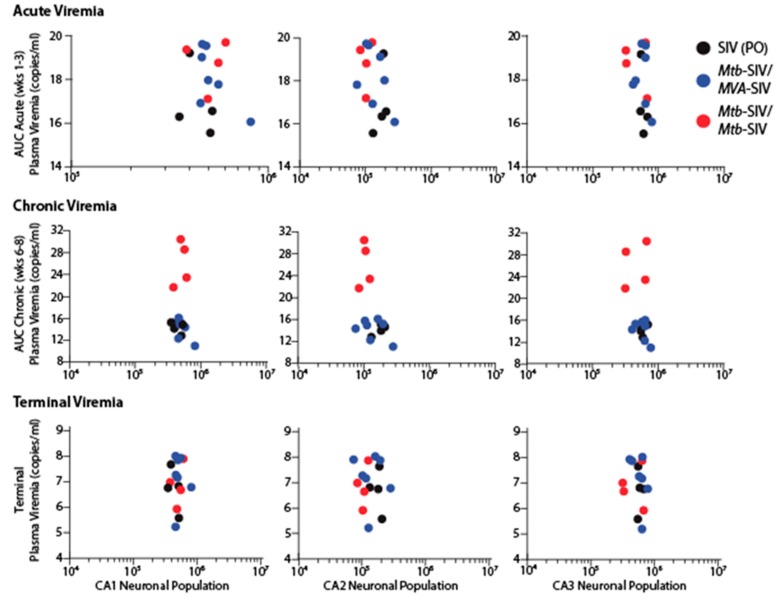
Neuronal population correlation. Despite a naturally occurring biological variance in viral loads, there was a lack of correlation between neuronal populations and plasma viral loads. AUC: area under the curve. SIV: simian immunodeficiency virus, Mtb-SIV: *Mycobacterium tuberculosis*-SIV vaccine, MVA-SIV: Modified vaccinia virus Ankara-SIV, CA: cornu ammonis

**Figure 5:**
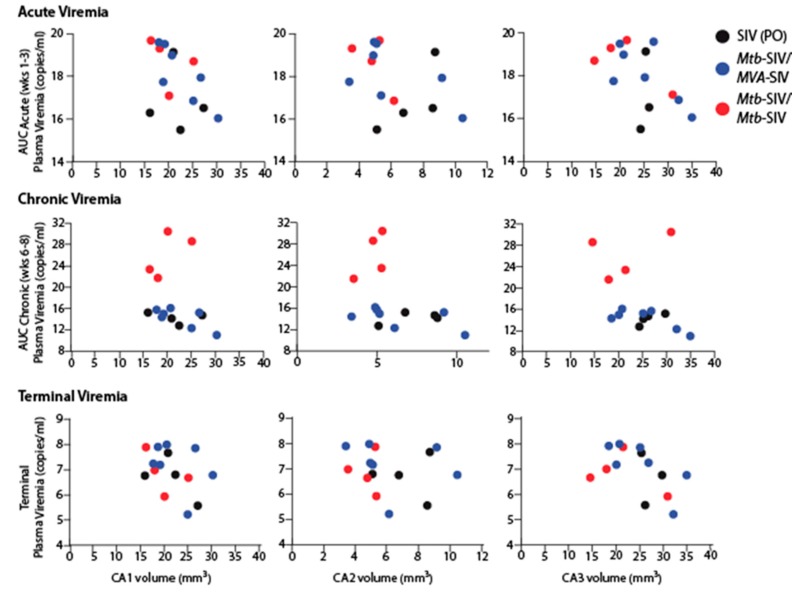
Volume correlation. There was a lack of correlation between regional volume and plasma viral loads.

The changes in neuronal frequencies and volumes between SIV-naïve and SIV-infected animals were not directly correlated to plasma viremia at the time of euthanasia (Figure 4 and Figure 5). Similar results were obtained when we tested for a correlation between CSF viremia and neuronal frequencies and volumes. As previous studies have shown that pathogenesis outcome is dependent on peak and set-point viremia [57,58], we also tested for a potential correlation between acute and early chronic viremia, as assessed by area-under-the-curve analysis (AUC), with neuronal changes. The degree of neuronal frequency reduction in SIV-infected macaques was not associated with acute, chronic or terminal plasma viremia (Figure 4). Likewise, the volume of the subregions was not correlated with acute, chronic or terminal plasma viremia (Figure 5). 

## 4. Discussion

We previously reported significant pyramidal neuronal loss in the CA1–3 fields of the hippocampus following IV inoculation with SIVmac251 within 72 h after birth with an average infection time of 8.3 weeks [49]. Here, we expanded on these data and tested the effect of oral SIV infection on neuronal damage. The oral route of SIV infection better represents HIV-1 acquisition by breastfeeding, the most common route of new mother-to-child transmissions of HIV-1. Furthermore, the age period of 9–23 weeks is more representative of the time of breastfeeding or mixed feeding practices in the clinical setting [13,14]. 

Our data suggests a particular vulnerability of the CA1 and CA2 region of the hippocampus. While both IV and PO SIV-infected infant macaques displayed significant reductions in pyramidal neuronal populations in the CA1-3 fields compared to controls, this effect appeared to be even more pronounced in the orally-infected infants compared to the perinatally IV-infected subjects. However, this difference was not significant and should not be over-interpreted as infants in these two groups differed not only in age at the time and route of SIV infection, but also in the duration of SIV infection. Thus, future studies should determine whether increased viral exposure time is associated with a progressive neuronal loss. 

Studies of neonatal rodent models of intracranial HIV-1 tat antigen administration support clinical evidence that the neurons of the hippocampus, and hippocampal neurogenesis, are specifically susceptible to the neurotoxic cascade of HIV-1 proteins [40]. Upon intracranial injections of tat and gp-120 in neonatal rats, there is a reduction of neuronal populations in the CA1, CA2 and CA3 fields, delays in eye opening and reflex development, and altered prepulse inhibition, which are correlated to a decline in spatial memory [40,41]. In the current investigation, we examined a narrow parameter of hippocampal integrity, neuronal population and volume. With the decreased volumes of the CA1–2 regions and the reduced neuronal population in CA1–3 in all SIV-infected infants (Groups 1-2A–C), it would be expected that changes in neural circuitry extend beyond the hippocampus. Clinical data indicates significant deficits in working memory and executive functioning [28,59], which would indicate a disrupted hippocampal-prefrontal circuitry [60,61,62].

Much of pre-existing clinical research focuses on global cortical changes and the basal ganglia [23,31,63], but there is increasing evidence of HIV-1 related toxicity in the hippocampus [49]. Previously, neural atrophy of the hippocampus has been reported in young adult monkeys following SIV infection [64]. CA1 pyramidal neurons are probably the most studied class of neuron in the brain, and better understood from a structural and functional standpoint compared to other types of neurons in the hippocampus [65]. This is due to relative ease of obtaining intracellular recordings and field potential recordings from these regions [65]. Despite our findings of a decrease in hippocampal neurons of SIV-infected subjects, a direct connection between neuronal populations and viral load in plasma or CSF at the time of brain collection could not be established. 

Clinical studies have shown that HIV RNA levels are extremely high during infancy and early childhood, in comparison to adults [66]. A study found a trend in the mean HIV-1 RNA load rising from low values (<10,000 copies/mL) at birth, to extremely high (>100,000 copies/mL) within the first 24 months of life, and then decreasing very slowly until the end of those 24 months [67]. This same study compared the viral load of infants with early (in utero transmission) vs. perinatal infection and discovered significantly varying median HIV-1 RNA values during the early months of life. At birth these values were 10,800 copies/mL, and less than 400 copies/mL; and after one month of life these values were 716,000 copies/mL and 100,000 copies/mL; with peak values of 780,000 copies/mL and 243,000 copies/mL, respectively [67]. Data from a perinatal cohort demonstrated a strong connection between high HIV RNA viral load during early infancy and poor clinical outcome, and also found that children with a higher viral load experienced rapid progression of the disease compared to children with lower loads [67]. Similar correlations between plasma viral RNA levels and disease-free survival have also been described in SIV-infected infant macaques [68,69].

Although HIV-1 has not been shown to infect neurons, there is evidence that the virus exerts its actions indirectly on neurons [70]. There is also evidence that HIV proteins are responsible for disrupting ion homeostasis and depolarizing neurons; while other proteins are restricted to neurons to disrupt neuronal membranes or induce apoptosis of the proteins [71]. Indirect neurotoxicity of HIV may involve the activation of glial cells, which have shown to result in the production and release of inflammatory cytokines like tumor necrosis factor (TNF)-alpha [70,72]. Our finding that infant macaques in Group 2C had the most severe neuronal reduction in CA2 is consistent with this conclusion. We have previously shown that vaccination with *Mtb*-SIV caused persistent systemic immune activation prior to and post-SIV infection [51]. Although the prior study did not analyze brain inflammation, mycobacteria-induced immune activation was persistent and systemic, and thus, could have affected the brain as well [51]. Astrocytes, microglia and macrophages are significant HIV-1 targets in the brain and research suggests that indirect mechanisms could, in part, account for severe neuronal damage observed in HIV-positive patients [73,74,75]. 

## 5. Conclusions

The loss of hippocampal neurons may contribute to the rapid neurocognitive decline associated with pediatric HIV infection. While each subfield showed vulnerability to SIV infection, the CA1 and CA2 subregions demonstrated a potentially increased vulnerability to SIV infection. These data underscore the need for early and continued ART as well as the development of therapeutics targeting the CNS [38].

## Figures and Tables

**Table 1:** Characteristics of subjects. SIV: simian immunodeficiency virus

Group	Subject ID	Gender	Vaccine	Age of SIV Infection	Age at Euthanasia	Total Infection Period	Plasma SIV Ribonucleic Acid (RNA) (Copies/mL) ^*^
1	41622 ^1^	M	none	1 week	10 weeks	9 weeks	160,000,000
1	41614 ^1^	F	none	1 week	7 weeks	7 weeks	240,000,000
1	41615 ^1^	F	none	1 week	10 weeks	10 weeks	650,000,000
2A	42376 ^2^	F	none	9 weeks	21 weeks	12 weeks	5,800,000
2A	42380 ^2^	F	none	17 weeks	27 weeks	10 weeks	6,400,000
2A	42386 ^2^	F	none	10 weeks	22 weeks	12 weeks	380,000
2A	42388 ^2^	F	none	13 weeks	25 weeks	12 weeks	46,000,000
2B	42944 ^2^	M	*Mtb*-SIV/MVA-SIV	9 weeks	33 weeks	24 weeks	170,000
2B	42958 ^2^	F	*Mtb*-SIV/MVA-SIV	15 weeks	33 weeks	18 weeks	6,000,000
2B	42949 ^2^	M	*Mtb*-SIV/MVA-SIV	9 weeks	21 weeks	12 weeks	18,000,000
2B	42899 ^2^	F	*Mtb*-SIV/MVA-SIV	9 weeks	20 weeks	11 weeks	84,000,000
2B	42929 ^2^	F	*Mtb*-SIV/MVA-SIV	10 weeks	20 weeks	10 weeks	99,000,000
2B	42906 ^2^	F	*Mtb*-SIV/MVA-SIV	9 weeks	22 weeks	13 weeks	73,000,000
2B	42937 ^2^	M	*Mtb*-SIV/ MVA-SIV	9 weeks	21 weeks	12 weeks	15,000,000
2C	42925 ^3^	F	*Mtb*-SIV/*Mtb* –SIV	10 weeks	22 weeks	12 weeks	4,700,000
2C	42943 ^3^	M	*Mtb*-SIV/*Mtb* –SIV	10 weeks	21 weeks	11 weeks	9,900,000
2C	42950 ^3^	M	*Mtb*-SIV/*Mtb* –SIV	9 weeks	19 weeks	10 weeks	77,000,000
2C	42918 ^3^	M	*Mtb*-SIV/*Mtb* –SIV	19 weeks	34 weeks	15 weeks	860,000
3	40967 ^4^	F	None	N/A	15 wks	N/A	N/A
3	40929 ^4^	M	None	N/A	16 wks	N/A	N/A
3	41656 ^4^	F	None	N/A	16 wks	N/A	N/A
3	41660 ^4^	F	None	N/A	16 wks	N/A	N/A

* Plasma and cerebrospinal fluid (CSF) SIV RNA levels at time of euthanasia. Group 2 was exposed, starting at 9 weeks of age, once weekly to SIVmac251 by the oral route until infection was verified [50,51]. ^1^ Jensen et al., 2013 [53], ^2^ Jensen et al., 2016 [50], ^3^ Jensen et al., 2017 [51], ^4^ Jensen et al., 2013 [53]. Mtb-SIV: *Mycobacterium tuberculosis*-SIV vaccine, MVA-SIV: Modified vaccinia virus Ankara-SIV.
